# Clinical Outcomes Using Mycophenolate and Tacrolimus for Graft-versus-Host Disease Prophylaxis in Patients Undergoing Allogeneic Stem Cell Transplant: A Single Institution Experience

**DOI:** 10.7759/cureus.6893

**Published:** 2020-02-05

**Authors:** Hamza Hashmi, Shruti Bhandari, Jugraj Dhanoa, Xiaoyong Wu, Shesh Rai, Lindsay Figg, Timothy Baize, Maxwell Krem, Mohamed Hegazi, Robert Emmons

**Affiliations:** 1 Department of Blood and Marrow Transplant and Cellular Immunotherapy, H. Lee Moffitt Cancer and Research Center, Tampa, USA; 2 Division of Medical Oncology and Hematology, University of Louisville, Louisville, USA; 3 Internal Medicine, University of Louisville, School of Medicine, Louisville, USA; 4 Bioinformatics and Biostatistics, University of Louisville, School of Public Health and Information Science, Louisville, USA; 5 Division of Clinical Oncology Pharmacy, University of Louisville Hospital, Louisville, USA; 6 Division of Hematology and Blood and Marrow Transplant, University of Kentucky, Lexington, USA; 7 Division of Blood and Marrow Transplant, University of Louisville, Louisville, USA

**Keywords:** graft versus host disease, allogeneic, stem cell transplant

## Abstract

For recipients of allogeneic hematopoietic stem cell transplant (HSCT), mycophenolate mofetil (MMF) plus tacrolimus combination is mostly used in reduced-intensity (RIC), and nonmyeloablative conditioning (NMAC) whereas methotrexate and tacrolimus combination is preferred in myeloablative conditioning (MAC). We present single institution outcomes in patients undergoing allogeneic HSCT with both MAC and NMAC/RIC regimen using MMF and tacrolimus for graft-versus-host disease (GVHD) prophylaxis. Data from all adult patients who underwent allogeneic HSCT from 2007 to 2017 was collected from Data Back to Centers web-based application of Center for International Blood and Marrow Transplant Research (CIBMTR). A total of 150 patients were included with the mean age of 46.9 years. For the patients who received MAC (n=109), the cumulative incidence of grade II-IV acute GVHD at day 100 was 37%, grade II-IV acute GVHD at one year was 51%, and chronic GVHD at one year was 38%. For the patients who received NMAC/RIC (n=41), the cumulative incidence of grade II-IV acute GVHD at day 100 was 31%, grade II-IV acute GVHD at one year was 28%, and chronic GVHD at one year was 36%. This institutional analysis shows that the combination of MMF and tacrolimus yields acceptable outcomes for the prevention of acute and chronic GVHD.

## Introduction

Graft-versus-host disease (GVHD) is a serious and challenging complication of allogeneic hematopoietic stem cell transplant (HSCT). The risk of mortality with acute GVHD is approximately 10-20% [1]. Since acute GVHD remains a major threat to a successful outcome after allogeneic HSCT and because treatment of acute GVHD can be challenging, the use of appropriate prophylaxis is of paramount significance in the care of these patients. Based on retrospective data the combination of tacrolimus and methotrexate has been found superior to cyclosporine and methotrexate [2]. However, methotrexate (MTX) is associated with significant side effects including severe mucositis, delays in neutrophil and platelet engraftment, and renal pulmonary and hepatic adverse effects. MTX also requires dose adjustment or even discontinuation for renal dysfunction due to prolonged elimination times and associated risk of increased toxicity. These adjustments may affect the efficacy of GVHD prophylaxis.

Mycophenolate mofetil (MMF) has been utilized to improve GVHD prophylaxis and reduce toxicity. MMF is an ester prodrug of the immunosuppressant mycophenolic acid, which inhibits inosine monophosphate dehydrogenase resulting in blockade of de novo purine synthesis, thereby limiting the proliferation of lymphocytes [3]. Based on clinical studies, a combination of cyclosporine and MMF is more effective than either agent alone. MMF in combination with cyclosporine when compared with MTX in combination with cyclosporine, is associated with significant benefits including less severe mucositis, shorter time to neutrophil and platelet engraftment, reduction in the use of total parenteral nutrition and narcotic analgesia, and shorter hospitalization times [4-6]. In addition, MMF and cyclosporine combination, when compared with MTX and cyclosporine combination, was associated with similar relapse, non-relapse mortality, and overall survival (OS) [6]. Incidence of grades II-IV acute GVHD varied between studies. In a study reported by Hamilton et al., combination MMF and cyclosporine, when compared with MTX and cyclosporine for patients undergoing myeloablative matched sibling donor transplant, had a similar relapse, non-relapse mortality and overall survival but a higher incidence of grade III-IV acute GVHD [4].

Tacrolimus and MMF have been found to be synergistic in preclinical models [5]. In addition, tacrolimus does not appear to interact adversely with the metabolism of MMF, whereas cyclosporine causes a decrease in the trough levels of its active metabolite, mycophenolic acid [6]. The combination of tacrolimus and MMF has been evaluated for acute GVHD prophylaxis in both adult and pediatric patient populations undergoing matched sibling donor allogeneic HSCT after myeloablative conditioning (MAC) and nonmyeloablative conditioning (NMAC) regimens [7, 8]. These single-arm phase II studies have shown that combination tacrolimus and MMF appears to have reasonable efficacy for acute GVHD prophylaxis and is also well tolerated when compared with other regimens [9, 10]. There is only one prospective randomized controlled trial that evaluated combination tacrolimus and MMF with tacrolimus and MTX [7]. There were no significant differences in the incidence of relapse, non-relapse mortality, or overall and relapse-free survival. MMF was associated with less early toxicity than MTX but was not as effective in preventing severe acute GVHD, especially in unrelated donor transplants. Remaining outcomes, including relapse, non-relapse mortality, and overall survival, were similar between the two groups. A recent publication by Chhabra et al. evaluating outcomes in patients undergoing allogeneic HSCT with reduced intensity (RIC) comparing calcineurin inhibitor (CNI)-MMF and CNI-MTX based regimens reported similar outcomes with matched sibling donors but higher acute GVHD with CNI-MMF combination for unrelated donors (URD) [8]. Similarly, an abstract by Hamilton et al. reported outcomes in patients undergoing myeloablative HSCT from human leukocyte antigen matched sibling donor and matched URD using a CNI+MMF versus CNI + MTX for GVHD prevention from 2000-2013 [11]. The analysis revealed significantly worse acute GVHD and survival outcomes with cyclosporine + MMF compared to tacrolimus + MTX. In this study, we report clinical outcomes in patients who underwent allogeneic HSCT and received tacrolimus and MMF for GVHD prophylaxis.

## Materials and methods

Patients and data collection

Center for International Blood and Marrow Transplant Research (CIBMTR) has a web-based application called Data Back to Centers that allows centers to access their own previously submitted data. Using this application, we downloaded de-identified data on patients who underwent allogeneic HSCT from 2007 to 2017 at the University of Louisville Hospital. All adults above the age of 18 years who received MMF with tacrolimus for GVHD prophylaxis were included.

Data collected included age, gender, ethnicity, disease, conditioning regimen, donor type, stem cell source, the incidence of acute GVHD at day 100 and at one-year, chronic GVHD at one year, and survival status. Patients with missing survival status at one year were excluded. This study included only de-identified patient information; therefore, it was exempt from the institutional review board.

Statistical analysis

The simple and multiple logistic regression analyses were used to evaluate predictors for grade II-IV acute GVHD at day 100, where the potential predictors included age, gender, race, disease, conditioning regimen, donor type, and stem cell source. The estimates of the odds ratio and 95% confidence interval (CI) are reported. Survival curves were generated using the Kaplan-Meier method. Statistical significance was considered for p < 0.05. Data analysis was performed by means of SAS v9.4 software (SAS Institute, Cary, NC, USA). 

## Results

A total of 150 patients were included in the analysis. Patients’ demographic and transplant characteristics are shown in Table 1. Most patients were females (52%) and Caucasian (86%). Acute myelogenous leukemia was the most common diagnosis (53%). One hundred and nine out of 150 (72%) patients received MAC regimen.

**Table 1:** Demographic and clinical characteristics of patients MAC - myeloablative conditioning; NMAC - nonmyeloablative conditioning; RIC- reduced-intensity conditioning; AML- acute myeloid leukemia; ALL- acute lymphoblastic leukemia; CML- Chronic myeloid leukemia; MDS/MPN - myelodysplastic syndrome/myeloproliferative neoplasm; GVHD - graft-versus-host disease

Variable	MAC (n=109)	NMAC/RIC (n=41)
	N (%)	N (%)
Age, years (mean)	46.7	52
Gender		
Male	49 (44)	23 (56)
Female	60 (55)	18 (44)
Race		
White	93 (85.3)	35 (85.4)
Black	13 (11.9)	4 (9.7)
Asian	3 (2.8)	2 (4.9)
Disease		
AML	63 (57.7)	17 (41.5)
ALL	19 (17.3)	2 (4.8)
CML	12 (11)	1 (2.4)
MDS/MPN	5 (4.8)	13 (31.7)
Other	10 (9.2)	8 (19.5)
Donor		
Matched Related	38 (34.8)	11 (26.8)
Matched/mismatched unrelated	67 (61.5)	27 (65.9)
Mismatched related	3 (2.8)	3 (7.3)
Multiple donor	1 (0.9)	0
Transplant source		
Bone marrow	7 (6.4)	6 (14.6)
Peripheral blood	92 (84.4)	32 (78)
Cord blood	10 (9.2)	3 (7.4)
Acute GVHD day 100 (total 125)		
None	42 (46.7)	19 (54.3)
Low grade (grade 1)	15 (16.6)	5 (14.3)
High grade (grade 2+3+4)	33 (36.7)	11 (31.4)
Missing	19	6
Acute GVHD year 1 (total 145)		
None	37 (35.2)	17 (42.5)
Low grade (grade 1)	15 (14.3)	12 (30)
High grade (grade 2+3+4)	53 (50.5)	11 (27.5)
Missing	4	1
Chronic GVHD year 1 (total 140)		
None	53 (52.5)	15 (38.5)
Limited	10 (9.9)	10 (25.6)
Extensive	38 (37.6)	14 (35.9)
Missing	8	

For the entire cohort, the cumulative incidence of grade II-IV acute GVHD at day 100 was 35%, grade II-IV acute GVHD at one year was 43%, and chronic GVHD at one year was 37%. In simple and multiple logistic regression analyses, none of the factors evaluated were related to the development of grade II-IV acute GVHD at day 100 (see Table 2).

**Table 2:** Simple and multiple logistic regression analyses for high-grade acute GVHD at day 100 MAC - myeloablative conditioning; NMAC - nonmyeloablative conditioning; RIC- reduced-intensity conditioning; AML- acute myeloid leukemia; ALL- acute lymphoblastic leukemia; CML- Chronic myeloid leukemia; MDS/MPN - myelodysplastic syndrome/myeloproliferative neoplasm; GVHD - graft-versus-host disease

	Univariable			Multivariable		
Predictors	Odds Ratio	95% CI	p-value	Odds Ratio	95% CI	p-value
Age	1	(0.97, 1.02)	0.733			
Gender						
Male	1					
Female	1.55	(0.73, 3.27)	0.251			
Race						
White	1					
Black	1.87	(0.61, 5.72)	0.276			
Disease						
AML	1			1		
ALL	0.51	(0.15, 1.72)	0.277	0.39	(0.08, 2.04)	0.267
CML	4.46	(1.05, 18.91)	0.042	1.09	(0.19, 6.30)	0.920
MDS/MPN	1.06	(0.32, 3.54)	0.921	2.49	(0.29, 21.59)	0.409
Other	0.96	(0.29, 3.13)	0.941	1.18	(0.18, 7.92)	0.867
Conditioning regimen						
MAC	1					
NMAC/RIC	0.79	(0.34, 1.82)	0.582			
Donor						
Related	1					
Unrelated	1.26	(0.59, 2.72)	0.551			
Transplant source						
Peripheral blood	1					
Bone marrow	0.65	(0.16, 2.60)	0.544			
Cord blood	0.74	(0.18, 3.05)	0.682			

**Figure 1:**
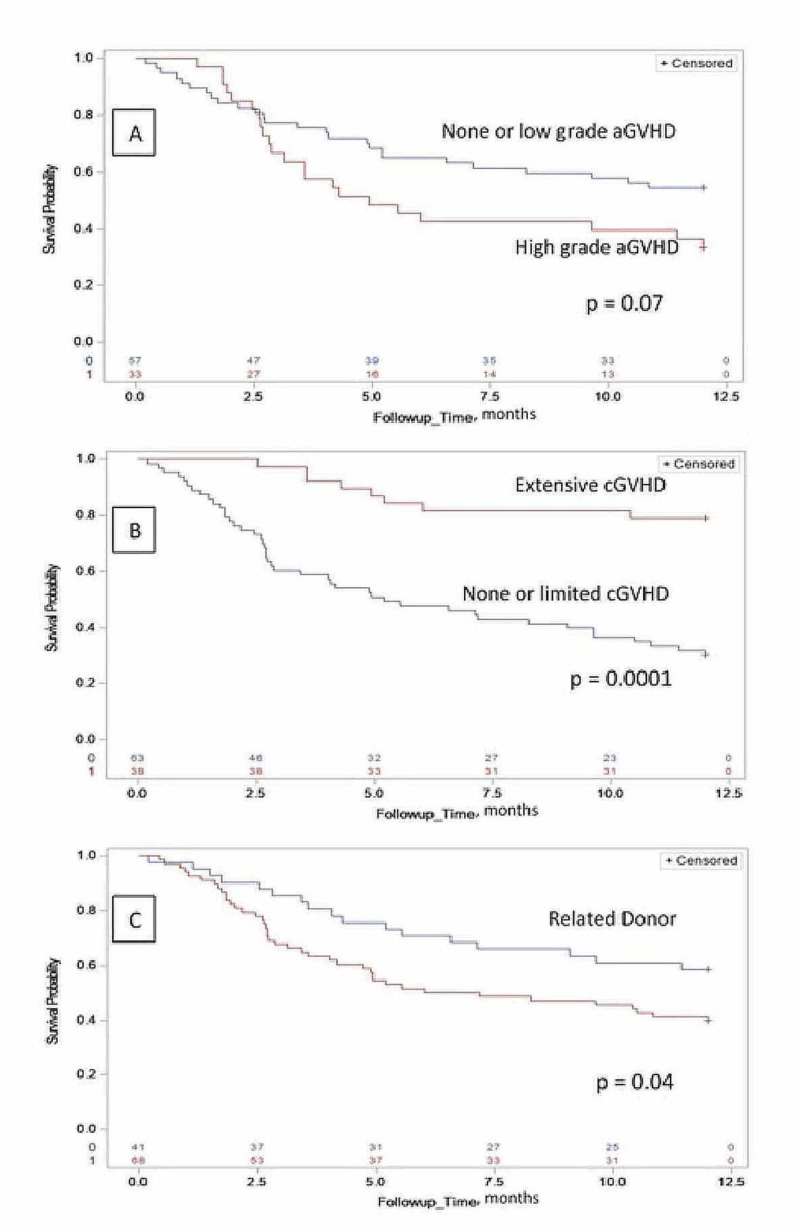
Corresponds to patients who received myeloablative conditioning regimen for allogenic hematopoietic stem cell transplant. A: The Kaplan-Meier estimate of the one-year overall survival (OS) time based on acute GVHD at day 100 (p-value based on the log-rank test is 0.07). B: The Kaplan-Meier estimate of the one-year OS time based on chronic GVHD (p-value on the log-rank test is 0.0001). C: The Kaplan-Meier estimate of the one-year OS time based on donor status (p-value based on the log-rank test is 0.04). aGVHD - acute graft versus host disease; cGVHD - chronic graft versus host disease

**Figure 2:**
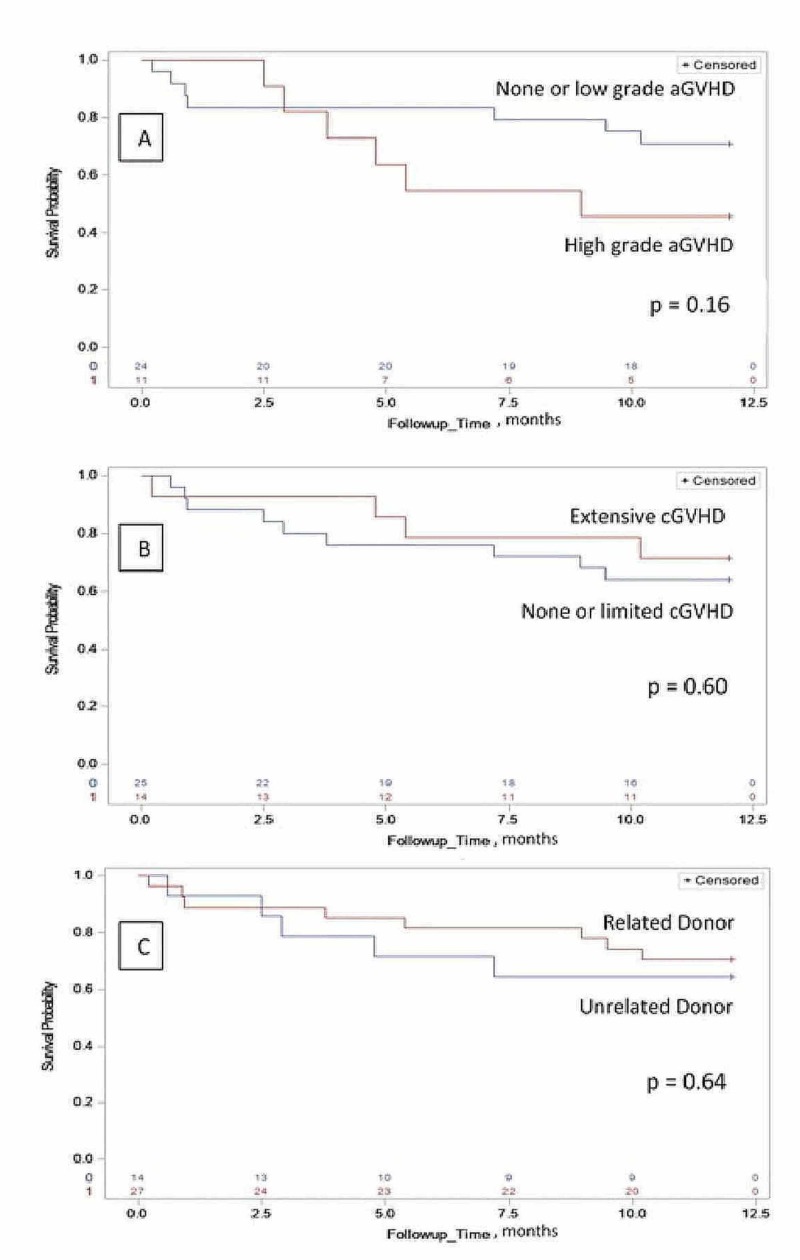
Corresponds to patients who received non-myeloablative or reduced-intensity conditioning regimen for allogenic hematopoietic stem cell transplant A: The Kaplan-Meier estimate of the one-year overall survival (OS) time based on acute GVHD at day 100 (p-value based on the log-rank test is 0.16). B: The Kaplan-Meier estimate of the one-year OS time based on chronic GVHD (p-value on the log-rank test is 0.60). C: The Kaplan-Meier estimate of the one-year OS time based on donor status (p-value based on the log-rank test is 0.64) aGVHD - acute graft versus host disease; cGVHD - chronic graft versus host disease

For the patients who received MAC regimen, the cumulative incidence of grade II-IV acute GVHD at day 100 was 37%, grade II-IV acute GVHD at one year was 51%, and chronic GVHD at one year was 38% (Figure 1). For the patients who received NMAC/RIC regimen, the cumulative incidence of grade II-IV acute GVHD at day 100 was 31%, grade II-IV acute GVHD at one year was 28%, and chronic GVHD at one year was 36% (Figure 2). 

**Figure 3:**
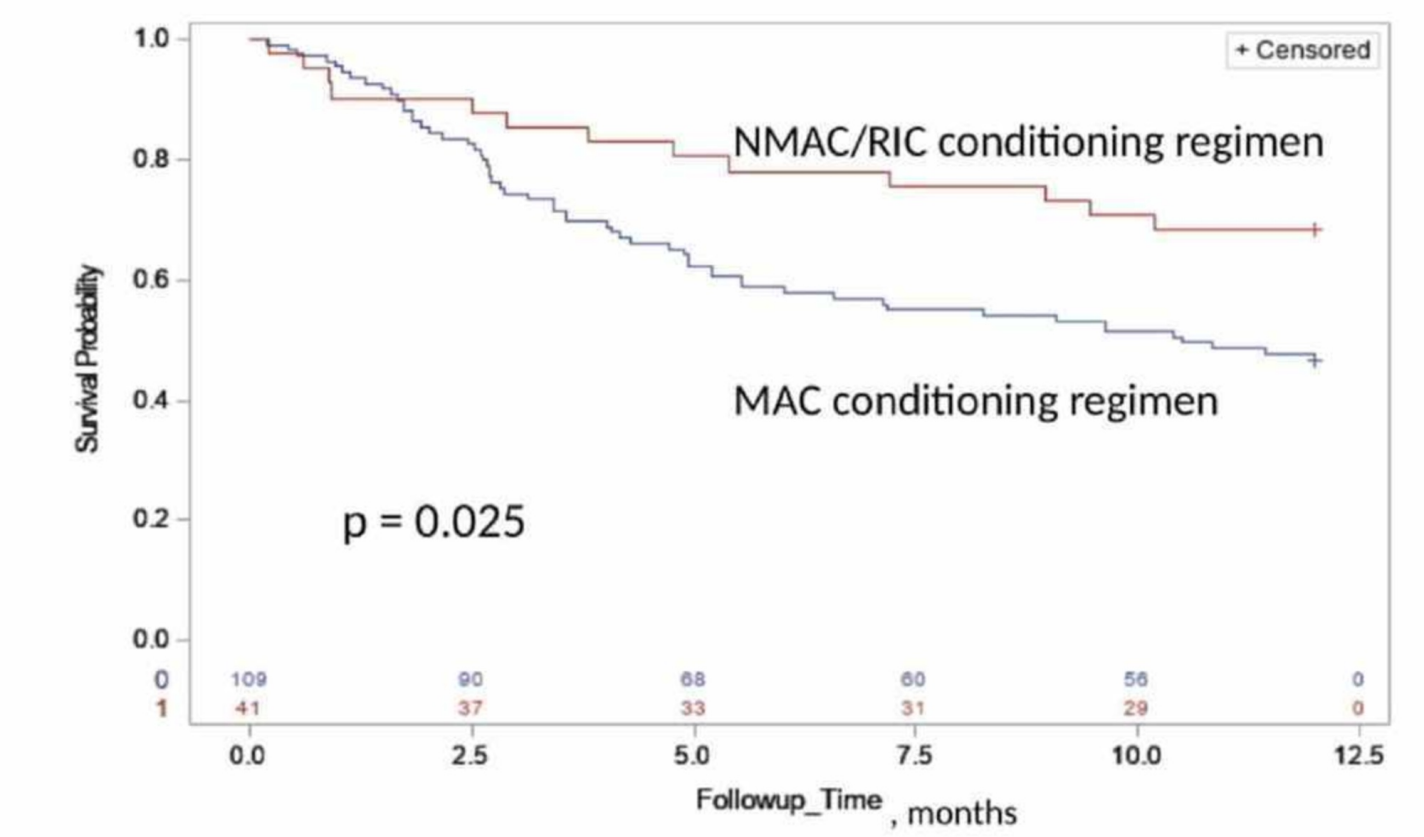
The Kaplan-Meier estimate of the one-year overall survival time based on conditioning regimen group The Kaplan-Meier estimate of the one-year overall survival (OS) time based on the conditioning regime group (p-value based on the log-rank test is 0.034). MAC - myeloablative conditioning; NMAC/RIC - nonmyeloablative conditioning/reduced-intensity conditioning

One-year survival rate for the entire cohort, patient receiving MAC regimen and NMAC/RIC regimen were 53% (45%, 61%), 47% (37%, 56%) and 68% (54%, 83%), respectively (Figure 3). The one-year survival was significantly associated with the conditioning regimen (p = 0.025), favoring better survival with the white race and NMAC/RIC regimen (Figure 2 and 3). For patients receiving MAC regimen, the median overall survival was 10.5 months and significantly associated with chronic GVHD (p < 0.0001) and donor status (p = 0.04), favoring better survival in patients with extensive chronic GVHD and related donor transplant (Figure 1).

## Discussion

Adequate GVHD prophylaxis plays a major role in patients undergoing allogeneic HSCT. Our study shows that the combination of tacrolimus and MMF is an option with reasonable efficacy for GVHD prophylaxis in these patients. Only one randomized controlled trial is reported in the literature as of this date that compares combination tacrolimus and MMF to combination tacrolimus and MTX for acute GVHD prophylaxis. This trial included 42 patients undergoing allogeneic HSCT, with predominantly myeloablative (90%) conditioning and peripheral blood as the sole source of stem cells [7]. The recent major study by Chhabra et al. utilizing CIBMTR database evaluated outcomes in patients undergoing allogeneic HSCT with reduced-intensity conditioning comparing CNI-MMF and CNI-MTX based regimens. This analysis demonstrated equivalent outcomes in those with matched related donors using either of the four CNI-based combinations and inferior efficacy of MMF-based approach regarding grade II to IV acute GVHD and non-relapse mortality in those with URD [8]. The analysis did not suggest using a specific regimen in URD allogeneic HSCT recipients using RIC and peripheral blood graft based on the lack of significant differences in OS. An abstract by Hamilton et al. reported outcomes in patients undergoing myeloablative HSCT comparing CNI + MMF versus CNI + MTX for GVHD prophylaxis [11]. The analysis revealed significantly worse acute GVHD with cyclosporine + MMF compared to tacrolimus + MTX. In the URD cohort, tacrolimus + MMF (n=424) was associated with a higher incidence of chronic GVHD (HR 1.47, p < 0.001), and worse OS (HR 1.34, p = 0.001) compared to tacrolimus + MTX. As the study was presented in the form on an abstract, no data is available for review on the outcomes, including acute GVHD with tacrolimus + MMF in the matched sibling donor cohort.

There are several aspects of our study that have significant clinical relevance. The role of tacrolimus + MMF combination as an effective regimen for GVHD prophylaxis in NMAC/RIC HSCT is well established [8]. However, in our study majority of the patients received MAC which has not been as studied as robustly in literature. Similarly, our study included both matched related and unrelated donors as a source of stem cells. Based on the review of existing literature, this is the largest single-institution study that evaluated acute GVHD prophylaxis with tacrolimus and MMF in patients undergoing allogeneic HSCT.

The primary outcomes reported in our study were similar to those reported in other historical data. For patients receiving MAC, the incidence of grade II-IV acute GVHD at day 100 was 37% in our study. Incidence of grade II-IV GVHD was reported as high as 78% with MTX + tacrolimus arm and 79% with MMF + tacrolimus arm by Perkins et al. Similarly, for the MAC group, the incidence of one-year extensive chronic graft-versus-host disease was 38% in our study versus 62% in MMF + tacrolimus arm and 73% in MTX + tacrolimus arm in the study by Perkins et al. [7]. One-year survival rate was 53% in our study versus 42% in MTX + tacrolimus arm and 54% MMF + tacrolimus in arm in the study by Perkins et al. and 60% in MTX + tacrolimus and 55% MMF + tacrolimus arm in the study by Chhabra et al. [7, 8].

Based on our results, MMF and tacrolimus combination had reasonable efficacy as a prophylactic regimen for GVHD. Historical data has shown that MMF and tacrolimus combination is associated with a lower incidence of mucositis, delayed platelet recovery, and the need for narcotic analgesia [4, 6, 12]. Due to the nature of the registry data, these outcomes could not be evaluated. Although no statistical significance was achieved, our study did reveal that patients with a higher grade of acute GVHD had worse one-year OS, likely related to the mortality with acute GVHD (Figure 1A). On the other hand, patients with extensive chronic GVHD had a better one-year OS (Figure 1B). This was perhaps due to a better graft-versus-malignancy effect. Long term morbidity and mortality from chronic GVHD could not be assessed. Patients receiving stem cells from related donors have better survival than those with URD as a source of stem cells. The degree of human leukocyte antigen (HLA) disparity as a predictor of long-term survival has been reported in the literature [12]. When comparing survival outcomes between MAC and NMAC/RIC regimen, the study showed that patients receiving MAC regimen had worse one-year OS (Figure 3). It has been well established that patients receiving MAC regimen have higher non-relapse or transplant-related mortality and lower incidence of relapse. Due to limitations from using registry data, it is not possible to determine if the higher mortality was related to relapse or acute GVHD. 

Although this is a small study that represents outcomes from a single institution, it helps eliminate bias from variation in institutional practices related to the intensity of conditioning regimen, infectious prophylaxis, and supportive measures. Due it is a retrospective nature where de-identified data was downloaded from CIBMTR application called Data Back to Centers, it lacks information on important safety and efficacy endpoints evaluated in other studies, including the incidence of mucositis, renal impairment, T-cell recovery and timing of engraftment. No data could be extracted for the incidence of relapse, non-relapse mortality, grade III-IV acute GVHD, or GVHD relapse-free survival. The role of anti-thymocyte globulin (ATG) for acute GVHD prophylaxis has been well established in the literature, and ATG has been used at our institution; none of the patients in our study population from 2007-2017 received ATG [13]. As there is no comparator arm, outcomes were compared with the limited historical data available in the literature. This adds some degree of bias to the conclusions drawn from this study.

## Conclusions

In conclusion, this institutional analysis shows that for patients undergoing HSCT with MAC, combination of MMF and tacrolimus yields acceptable outcomes for prevention of acute and chronic GVHD as well as one-year OS. The results of this study need to be validated in a large, multicenter randomized controlled trial.
